# How legal motivation buffers the effects of moral disengagement on school bullying among Chinese college students

**DOI:** 10.3389/fpsyg.2025.1583706

**Published:** 2025-07-11

**Authors:** Jianhua He, Zhiqiang Wang, Shuhui Xu

**Affiliations:** ^1^School of Mechanical Engineering, Tongling University, Anhui, China; ^2^School of Teacher Education, Taizhou University, Zhejiang, China; ^3^Institute of Higher Education, Wenzhou University, Zhejiang, China

**Keywords:** moral disengagement, school bullying, legal motivation, legal education, legal socialization

## Abstract

**Background:**

Grounded in Bandura’s social cognitive theory and the risk-buffering model, this study investigates how moral disengagement and legal motivation jointly influence school bullying among Chinese university students.

**Methods:**

A cross-sectional survey was administered to 409 students across mainland China. Confirmatory factor analysis established measurement validity. Pearson’s correlations and independent-samples t-tests assessed bivariate relationships, and moderation analysis using Hayes’ PROCESS macro tested whether legal motivation buffered the effect of moral disengagement on bullying.

**Results:**

Moral disengagement correlated positively with bullying perpetration, while legal motivation correlated negatively. Moderation analysis revealed that higher levels of legal motivation attenuated the positive link between moral disengagement and school bullying.

**Conclusion:**

Enhancing legal motivation may mitigate the influence of moral disengagement on bullying. Integrating legal-education initiatives with moral development interventions could therefore offer a more effective strategy for reducing school bullying.

## 1 Introduction

Bullying—deliberate, repeated aggression by an individual or group more powerful against someone in a vulnerable position—remains a pervasive problem in schools worldwide. Recent research has highlighted that bullying significantly affects the quality of life and mental health of many adolescents (Wang et al., 2019). It is not only a critical social issue during school years but also a strong predictor of antisocial behavior and violent crime in adolescence and early adulthood. For instance, Olweus (2013) found that among bullies in grades 7–10, 55% engaged in at least one criminal act between the ages of 16 and 24, while 36% committed three or more criminal offenses. Victims and perpetrators alike experience enduring mental health problems, social difficulties, and an elevated risk of later antisocial or criminal behavior (Cosma et al., 2020; Farrington and Ttofi, 2011; Kim et al., 2006; Lösel and Bender, 2003; Walters and Espelage, 2019).

In China, the severity of this issue was tragically underscored on March 10, 2024, in Feixiang District, Handan (Hebei Province), when a 13-year-old student’s body was discovered in a vegetable greenhouse at the hands of three underage classmates (Li, 2025). National surveys confirm its ubiquity: the China Youth Research Center’s “Research on Adolescent Legal Education” project found that 53.5 percent of 3,108 underage students had experienced bullying (Li, 2024), and a large-scale study of 3,042 college students reported a significant association between bullying experiences and emotional disorders, even after controlling for demographic variables (Wang et al., 2023). Similarly, the 2017 death of a cadet at the Malaysian National Defence University highlights the equally tragic outcomes of peer bullying beyond China’s borders.

Drawing on Bandura’s social-cognitive theory of moral disengagement and Tyler’s self-determination–based theory of legal motivation, this interdisciplinary study examines two psychological mechanisms that may drive bullying involvement and its prevention. Moral disengagement—cognitive distortions such as moral justification, diffusion of responsibility, and dehumanization—enables individuals to behave aggressively without guilt (Bandura, 1999, 2016; Yang et al., 2010) and has been robustly linked to higher rates of bullying (Caravita et al., 2012; Thornberg and Jungert, 2013; Wang et al., 2012; Xie et al., 2023). In contrast, legal motivation—the intrinsic drive to learn, uphold, and internalize legal norms such as freedom, order, and equality—promotes voluntary compliance and discourages rights-violating behavior (Tyler, 2003, 2006; Trinkner et al., 2018; Xu and Yan, 2022).

Although prior research has independently linked moral disengagement and legal motivation to aggressive or rule-compliant behaviors, no study has tested whether legal motivation can buffer the effect of moral disengagement on bullying—particularly within the unique social and institutional context of Chinese universities. By integrating these legal-psychological perspectives, our research makes two original contributions: first, it provides the first empirical evidence that legal motivation attenuates the positive relationship between moral disengagement and school bullying among Chinese university students; second, it advances an interdisciplinary framework that bridges legal education and moral-development interventions, offering novel insights for designing more effective bullying-prevention strategies.

Building on these contributions, the present study addresses two primary objectives: (1) to assess the extent to which moral disengagement predicts engagement in school bullying among Chinese university students, and (2) to examine how legal motivation moderates this relationship. In doing so, we aim to inform the development of integrated interventions that combine legal-educational initiatives with efforts to strengthen moral self-regulation, thereby reducing both immediate harm and long-term antisocial outcomes.

## 2 Theoretical framework

### 2.1 Moral disengagement and school bullying

Bandura introduced the psychological concept of moral disengagement to explain how individuals can maintain a positive self-image even after harming others (Bandura, 1999; Bandura, 2016). Moral disengagement refers to a cognitive distortion mechanism through which individuals justify, rationalize, or reinterpret unethical or harmful behaviors, thereby interfering with their moral self-regulation and self-evaluation. This mechanism enables individuals to accept, or even deliberately engage in, such behaviors without perceiving them as wrongful or experiencing feelings of guilt or remorse. In other words, moral disengagement represents a failure of internal moral standards, allowing individuals to reconstruct immoral behaviors as morally acceptable while minimizing the sense of responsibility for the consequences of these behaviors (Yang et al., 2010). This mechanism encompasses eight specific forms, including moral justification, euphemistic labeling, diffusion of responsibility, distortion or disregard of harmful consequences, dehumanization, and blaming the victim (Bandura, 1999).

Based on social cognitive theory (Dodge and Coie, 1987), an individual’s moral cognition of violent behaviors directly influences their behavioral manifestations. Specifically, individuals with higher levels of moral disengagement tend to rationalize aggressive behaviors, thereby further increasing the likelihood of engaging in such behaviors (Xie et al., 2023). Research has demonstrated that the mechanism of moral disengagement is closely associated with various roles in bullying behavior (Caravita et al., 2012). A meta-analysis further identified moral disengagement as a significant predictor of cyberbullying (Mishna et al., 2014). Additionally, moral disengagement has been significantly linked to more pro-bullying behaviors and fewer defensive actions among bystanders (Thornberg and Jungert, 2013). In summary, extensive research consistently indicates a significant positive correlation between levels of moral disengagement and aggressive behaviors (Wang et al., 2012; Shaikh et al., 2020).

Drawing on the foregoing theoretical considerations and empirical evidence, we propose the following hypothesis:

H1: It is hypothesized that university students’ levels of moral disengagement are positively associated with their engagement in school bullying behaviors.

### 2.2 Moderating role of legal motivation

Legal motivation refers to individuals’ intrinsic drive to understand, uphold, and practice the law, rooted in the internalization of its core values such as freedom, order, justice, and equality (Tyler, 2006; Xu and Yan, 2022). It reflects not only cognitive recognition of legal norms but also affective and motivational alignment with the rule of law. Legal motivation emerges when individuals perceive legal authority as legitimate, morally aligned, and beneficial to personal and collective wellbeing (Trinkner et al., 2018). This internalized motivation guides behavior not out of fear of sanctions, but from a value-based commitment to lawful conduct and social harmony (Tyler, 2003; Tyler and Bies, 1990).

From the perspective of legal socialization, adolescents’ beliefs about law and authority strongly influence their behavioral choices. Research shows that adolescents who perceive legal authorities as fair and legitimate are more likely to conform to legal norms, while those with low legitimacy perceptions exhibit higher rates of delinquency and violence (Fagan and Tyler, 2005; Kaiser and Reisig, 2019; Nivette et al., 2020). Legal motivation, as an outcome of such perceptions, not only promotes compliance but also enables individuals to resist deviant tendencies, such as those stemming from moral disengagement.

Building on the social–cognitive theory (Bandura, 1999) and the Risk-Buffering Model (Hollister-Wagner et al., 2001), we propose that legal motivation moderates the relationship between moral disengagement and school bullying. Moral disengagement weakens moral self-regulation, allowing individuals to rationalize harmful conduct (Bandura et al., 1996). However, legal motivation, by reinforcing prosocial norms and rule-consciousness, may function as a protective factor that mitigates this effect.

Specifically, when legal motivation is high—indicating strong internalization of legal norms and sensitivity to legal consequences—individuals are less likely to engage in bullying even if they experience moral disengagement. In contrast, when legal motivation is low, moral disengagement may more readily translate into actual bullying behavior. This interaction effect reflects the boundary condition function of legal motivation and aligns with prior evidence on how internal motivations buffer cognitive and emotional risk factors (Eisenberg et al., 2002).

Building on the foregoing hypothesis and theoretical framework, we propose our second hypothesis:

H2: Legal motivation moderates the positive relationship between moral disengagement and school bullying, such that the association between moral disengagement and bullying behavior is weaker at higher levels of legal motivation.

## 3 Methods

### 3.1 Participants and data collection process

A total of 443 university students from mainland China were initially recruited via cluster sampling, using class units as the sampling frame, to complete an online questionnaire. After excluding incomplete, unengaged, or patterned responses, the final sample comprised 409 participants (mean age = 19.02, SD = 0.73; 136 males, 273 females; 173 freshmen, 107 sophomores, 114 juniors, 15 seniors), yielding an effective response rate of 92.33%.

The survey was administered through the Questionnaire Star platform and distributed via WeChat. Prior to data collection, informed consent was obtained from all participants. During scheduled class sessions, the primary investigator (a graduate student in psychology) introduced the study’s purpose, emphasized anonymity and confidentiality, and provided instructions on how to complete the items. The questionnaire took approximately 15 min to complete. Valid responses were then exported and entered into SPSS for analysis. This study protocol received approval from the Ethics Review Committee of the authors’ affiliated university.

### 3.2 Measures

#### 3.2.1 Moral disengagement

Participants’ levels of moral disengagement were assessed using the Moral Disengagement Scale developed by Bandura et al. and revised by Wang and Yang (2010), consisting of 32 items across eight dimensions (e.g., diffusion of responsibility, dehumanization). One sample item is: “Telling a small lie is not a big deal because it doesn’t really hurt anyone.” Responses were rated on a 5-point Likert scale from 1 (strongly disagree) to 5 (strongly agree), with higher scores indicating stronger moral disengagement. In the present study, the overall internal consistency coefficient of the scale was 0.935. The Cronbach’s α coefficients for the eight dimensions were as follows: 0.715 for moral justification, 0.695 for euphemistic labeling, 0.694 for advantageous comparison, 0.586 for displacement of responsibility, 0.720 for diffusion of responsibility, 0.688 for distortion of consequences, 0.697 for dehumanization, and 0.756 for attribution of blame. Confirmatory factor analysis showed good construct validity (χ^2^/df = 2.53, NFI = 0.93, GFI = 0.91, IFI = 0.99, CFI = 0.96, TLI = 0.95, RMSEA = 0.05).

#### 3.2.2 Legal motivation

Legal motivation was measured using the Legal Motivation Questionnaire for College Students (Xu and Yan, 2022), comprising 22 items across three dimensions: motivation to learn, obey, and use the law. A sample item is: “I study law to cultivate legal thinking. I believe it is necessary to cultivate the concept of ‘rule of law’.” Items were rated on a 5-point Likert scale from 1 (strongly disagree) to 5 (strongly agree), with higher total scores reflecting stronger legal motivation. The internal consistency coefficients were 0.909 (learn), 0.943 (obey), and 0.922 (use); overall reliability was 0.964. CFA results indicated good structural validity (χ^2^/df = 4.30, CFI = 0.91, TLI = 0.90, RMSEA = 0.09, SRMR = 0.04).

#### 3.2.3 School bullying

School Bullying. School bullying behaviors were measured using the Chinese version of the Olweus Bullying Questionnaire, revised by Zhang and Wu (1999). The scale includes 7 items covering four types of bullying: physical, verbal, relational, and cyberbullying. A sample item is: “I hit, kick, push, bump into, or threaten others.” Responses were rated on a 5-point Likert scale, and higher cumulative scores indicated higher levels of bullying behavior. Internal consistency was 0.924, and CFA results supported good structural validity (χ^2^/df = 4.91, NFI = 0.96, RFI = 0.94, IFI = 0.97, CFI = 0.97, TLI = 0.95, RMSEA = 0.08).

### 3.3 Statistical analysis

The primary statistical software used for this study was SPSS 25.0 and AMOS. Confirmatory factor analysis (CFA) was conducted to test the structural validity of each questionnaire. Harman’s single-factor test was used to assess common method bias. Pearson’s correlation was employed to explore the relationships between variables. Independent sample *t*-tests were performed to examine gender differences across the variables. Finally, the PROCESS macro was utilized to test the moderating effect of legal motivation in the relationship between moral disengagement and school bullying.

## 4 Results

### 4.1 Common method bias test

To address potential common method bias inherent in self-report data, we conducted Harman’s single-factor test. Exploratory factor analysis extracted 12 factors with eigenvalues greater than 1, and the first factor accounted for only 27.01% of the total variance—well below the 40% threshold—indicating that common method bias is unlikely to threaten our findings.

### 4.2 Descriptive and correlation analysis

Descriptive statistics and Pearson correlation analyses were conducted for the main variables. As shown in Table 1, moral disengagement was significantly positively correlated with school bullying *r* = 0.349, *p* < 0.01), indicating that higher levels of moral disengagement are associated with greater engagement in school bullying behaviors. This finding supports Hypothesis 1. In addition, legal motivation was significantly negatively correlated with school bullying (*r* = 0.−0.232, *p* < 0.01), suggesting that individuals with higher legal motivation are less likely to engage in bullying.

**TABLE 1:** Descriptive statistics and correlation analysis of the main variables.

Variables	1	2	3	4	5
Gender	1				
Academic year	0.009	1			
3.MD	0.206***	−0.038	1		
4. LM	−0.044	−0.125*	−0.368**	1	
5.SB	0.204***	0.078	0.349**	−0.232**	1
*M* ± *SD*			63.99 ± 18.71	90.81 ± 14.01	7.57 ± 2.45
*M* ± *SD (M)*			69.45 ± 21.29	89.94 ± 14.64	8.28 ± 3.71
*M* ± *SD (F)*			61.27 ± 16.67	91.25 ± 13.67	7.22 ± 1.35
T			3.92***	−0.87	3.23***

Gender (M = Male, F = Female); MD, moral disengagement; LM, legal motivation; SB, school bullying. *p < 0.05, **p < 0.01, ***p < 0.001.

Independent sample *t*-tests further revealed significant gender differences in moral disengagement and school bullying, with males scoring significantly higher than females on both variables. Moreover, grade level was significantly negatively correlated with legal motivation. Based on these results, gender and grade level were included as covariates in the subsequent moderation analysis.

### 4.3 The moderating effect of legal motivation on the relationship between moral disengagement and school bullying

To test Hypothesis 2, which proposed that legal motivation moderates the relationship between moral disengagement and school bullying, all variables were standardized to reduce multicollinearity. The moderation analysis results (see Tables 2, 3) showed that legal motivation had a significant negative moderating effect on this relationship (β = −0.31, *p* < 0.001).

**TABLE 2:** Analysis of the moderating effect of legal motivation.

Variables	School bullying		
	β	*t*	95%CI
Constants	−0.367	−3.621***	[−0.567, −0.168]
Gender	0.232	2.511*	[0.048, 0.410]
Academic year	0.091	2.039*	[0.003, 0.179]
Moral disengagement	0.239	5.095***	[0.148, 0.332]
Legal motivation	−0.132	−2.863***	[−0.221, −0.040]
Moral disengagement * Legal motivation	−0.311	−8.276***	[−0.385, −0.237]
*F*	31.621***		
*R* ^2^	0.282		

***p < 0.001, *p < 0.05.

**TABLE 3:** The moderating effect of legal motivation on moral disengagement on school bullying.

Variable	Moral disengagement	Effect	*t*	*SE*	95%CI
Legal motivation	*M*–1*SD*	0.551	9.697***	0.057	[0.439, 0.662]
Legal motivation	M	0.240	5.116***	0.047	[0.148, 0.332]
Legal motivation	*M* + 1*SD*	−0.071	−1.129	0.063	[−0.195, 0.053]

***p < 0.001.

**FIGURE 1:**
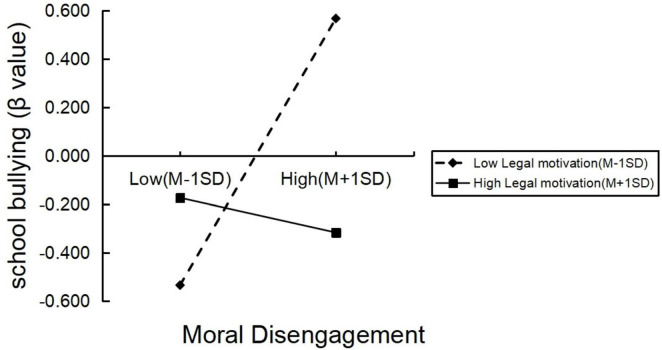
Moderating effect of legal motivation on moral disengagement and school bullying.

Simple slope tests were further conducted to explore the nature of the moderation effect. Participants were divided into high and low legal motivation groups. In the low legal motivation group, moral disengagement significantly predicted school bullying (simple slope = 0.55, *p* < 0.001). In contrast, the prediction was not significant in the high legal motivation group (simple slope = −0.07, *p* > 0.05). These results are illustrated in Figure 1 and indicate that as legal motivation increases, the positive relationship between moral disengagement and school bullying weakens. This finding supports Hypothesis 2 and suggests that legal motivation can buffer the negative effects of moral disengagement on bullying behavior among university students.

## 5 Discussion

Amid mounting concerns over the prevalence of school bullying and its detrimental effects on adolescent mental health and social development, the present study was undertaken to examine the role of moral disengagement in facilitating bullying behaviors and to elucidate the cognitive processes that underpin this relationship. Consistent with prior research (Montero-Carretero et al., 2021; Fu and Wu, 2014), our findings demonstrate that higher levels of moral disengagement significantly predict greater involvement in school bullying. Specifically, individuals who engage in moral disengagement employ strategies such as moral justification, euphemistic labeling, and displacement of responsibility to cognitively reframe harmful actions (Bandura, 1990, 2002). Drawing on social cognitive theory, we posit that these mechanisms serve to attenuate self-sanction, thereby lowering inhibitions against aggressive and bullying behaviors.

Our findings further demonstrate that the predictive effect of moral disengagement on school bullying is moderated by legal motivation. Specifically, in the low legal motivation group, moral disengagement strongly predicted school bullying, while in the high legal motivation group, this relationship was not significant. This result supports the view that legal motivation can buffer the influence of moral disengagement on deviant behavior, a mechanism also observed in international studies emphasizing the role of normative commitment in reducing aggression (Gini et al., 2011; Tapp and Kohlberg, 2010).

From a theoretical perspective, legal motivation may function as a form of internalized moral standard that competes with cognitive mechanisms of moral disengagement. Individuals with high legal motivation are more likely to internalize legal norms and values, enhancing their sensitivity to the moral and legal implications of their actions and thereby reducing the likelihood of engaging in bullying behaviors. This supports and expands upon Bandura’s (2002) assertion that the activation or inhibition of moral disengagement is influenced by personal values and social influences.

Social Identity Theory (Tajfel and Turner, 1986) offers an additional explanatory framework. When individuals perceive themselves as members of a group that values legal compliance and moral behavior, they are more likely to align their actions with group norms. Legal motivation may thus reflect not only personal attitudes but also identification with law-abiding social groups. This identification fosters normative conformity, diminishing the cognitive justifications that sustain moral disengagement. Prior research has shown that social identification with prosocial peer groups can reduce bullying behaviors and enhance adherence to moral norms (Pozzoli and Gini, 2010).

In comparing these findings with international literature, the buffering effect of legal or moral internalization is echoed in cross-cultural studies that link civic values and legal consciousness to reduced aggressive conduct (Obermann, 2011; Guerra et al., 2011). These results underscore the cross-national relevance of internal motivational factors in curbing youth aggression and reinforce the importance of legal socialization.

In summary, this study enriches the theoretical understanding of how internal motivational factors, such as legal motivation, can mitigate the negative effects of moral disengagement. These findings extend the literature by demonstrating that legal motivation operates not only as a behavioral constraint but also as a cognitive-emotional moderator of moral processes, with implications for both theory and intervention design. Enhancing legal motivation among youth may serve as a protective factor in educational settings, reducing bullying by weakening the justification mechanisms underlying such behaviors.

## 6 Implications and recommendations

### 6.1 Theoretical implications

This study underscores the moderating role of legal motivation in the relationship between moral disengagement and school bullying, highlighting the potential of legal awareness as a protective factor. While recent research has begun to examine the influence of teacher justice on bullying (Luo et al., 2024), few studies have directly addressed legal motivation in this context. By integrating legal (legal motivation) and psychological (moral disengagement) perspectives, this research offers a novel interdisciplinary framework for understanding school bullying. It extends the application of Social Identity Theory (Tajfel and Turner, 1986) by demonstrating that a strong legal identity reduces norm-violating behaviors. These findings contribute to the theoretical development of integrated moral and legal education and support interdisciplinary approaches to bullying prevention.

### 6.2 Practical implications

In practical terms, the study underscores the importance of strengthening legal education in schools to promote intrinsic legal motivation among students. As Yu and Han (2023) suggest, incorporating specific legal content—such as the Public Security Administration Punishments Law and the Criminal Law—into school curricula is essential for fostering students’ understanding of their rights and responsibilities under the law. Moreover, employing interactive pedagogical methods such as mock trials, case analyses, and role-playing can further enhance students’ engagement with legal principles and deepen their understanding of legal consequences. These methods not only make the learning process more engaging but also foster a proactive commitment to lawful behavior. Additionally, the results of this study emphasize the need to clarify the legal responsibilities of teachers and school administrators. Drawing on Tyler and Bies’ (1990) principle that legitimate authority promotes compliance, schools should codify educators’ roles in identifying and addressing bullying, ensuring that teachers and administrators are well-equipped to intervene effectively.

### 6.3 Policy recommendations

Based on the findings of this study, we recommend that schools establish a robust system for responding to bullying that includes clear protocols for reporting, investigating, and providing feedback on incidents. This “report–investigate–respond–feedback” protocol, along with the creation of a dedicated campus safety committee, will help ensure that bullying is promptly addressed, minimizing potential harm to students. In addition, Zhao and Zhang’s (2022) research on the influence of class teachers’ leadership behaviors suggests that training teachers in interpersonal and leadership skills—such as empathy, emotional stability, and motivational practices—can significantly enhance students’ engagement and trust in authority. By improving the classroom climate, these leadership qualities can bolster students’ moral self-regulation and legal motivation. Finally, an integrated approach to moral and legal education, building on China’s 2016 curriculum reform (Ministry of Education of the People’s Republic of China, 2016), can bridge the gap between internal values and external legal norms. Through cross-disciplinary case discussions, experiential learning activities, and community engagement, students can internalize principles of fairness and respect, strengthening both their moral self-regulation and intrinsic legal motivation (Aquino et al., 2009).

These recommendations aim to provide a more comprehensive approach to addressing school bullying, combining legal education with moral development to foster a safer, more respectful school environment.

## 7 Limitations and directions for future research

This study introduces the concept of legal motivation into the research on school bullying, offering a novel interdisciplinary perspective. However, several limitations should be acknowledged. First, based on person–situation interaction theory (Magnusson and Stattin, 1998), behavior is influenced by both individual traits and environmental factors. In this study, only gender and academic year were controlled for, while important contextual variables such as school climate, peer norms, and socioeconomic status were overlooked, which may impact the robustness of the results. Future research should include these factors to provide a more comprehensive understanding. Second, the cross-sectional design and reliance on self-reports limit causal inference and may introduce social desirability bias. Future studies should consider longitudinal, behavioral, or experimental designs and incorporate multi-informant data to improve reliability and validity. Lastly, while the proposed strategies are theoretically grounded, they lack empirical testing. Future research should evaluate their feasibility and effectiveness in real-world settings to better inform practical interventions.

## Data Availability

The original contributions presented in the study are included in the article/supplementary material, further inquiries can be directed to the corresponding authors.
